# Seroconversion and Prevalence of Hepatitis B Surface Antigen among Vaccinated Health Care Workers in Ashanti Region, Ghana

**DOI:** 10.1155/2023/2487837

**Published:** 2023-12-19

**Authors:** Michael Agyemang Obeng, Daniel Kobina Okwan, Ernest Adankwah, Pisco Kofi Owusu, Samuel Asante Gyamerah, Kluivert Boakye Duah, Ellis Kobina Paintsil

**Affiliations:** ^1^Kumasi Centre for Collaborative Research in Tropical Medicine, Kwame Nkrumah University of Science and Technology, Kumasi, Ghana; ^2^Department of Anatomy, Kwame Nkrumah University of Science and Technology, Kumasi, Ghana; ^3^Department of Medical Diagnostics, Kwame Nkrumah University of Science and Technology, Kumasi, Ghana; ^4^Medilab Diagnostic Services Limited, Kumasi, Ghana; ^5^Department of Statistics and Actuarial Sciences, Kwame Nkrumah University of Science and Technology, Kumasi, Ghana

## Abstract

**Background:**

Health care workers (HCWs) constantly stand at a high risk of exposure to the hepatitis B virus because of the nature of their work. Hence, it is mandatory for HCWs to undergo hepatitis B vaccination. However, most HCWs in Ghana do not check their HBsAb titre after completion of their primary vaccination. This study assessed the prevalence of HBsAg and the seroconversion rate among vaccinated health care workers in the Ashanti Region, Ghana.

**Materials and Methods:**

A semistructured open-ended questionnaire was pretested and administered to 424 HCWs. Two (2) ml of blood was drawn and qualitative analyses (HBsAg, HBsAb, HBeAg, HBeAb, and HBcAb) were done on the blood samples. Samples that tested positive to HBsAb were quantified using ELISA. Data obtained were analysed using GraphPad Prism 9.

**Results:**

Out of the 424 study participants, 271 (63.9%) were females and 153 (36.1%) were males. Seroconversion (≥1 mIU/mL) and seroprotection (≥10 mIU/mL) through vaccination only among study participants were 67.5% (*n*/*N*  = 286/424) and 58.0% (*n*/*N*  = 246/424), respectively. Prevalence of hepatitis B viral infection was 2.4% (*n*/*N*  = 10/424). Anti-HBc seropositivity was 13.2%, and anti-HBs seronegativity was 24.1%. 2.4% (*n*/*N*  = 10/424) of study participants were negative to HBsAg but positive to HBcAb. In addition, 8.5% (*n*/*N* = 36/424) of the study participants were seroprotected due to exposure and recovery from previous HBV infection. Age, the number of doses received, taking a booster dose, and keeping a vaccination record card were significant factors influencing seroconversion status.

**Conclusion:**

This study reaffirms the need for HCWs to undergo a supervised primary hepatitis B vaccination course. Postvaccination serological testing should be done for all HWCs to confirm immunity and reduce their chances of contracting HBV infection.

## 1. Introduction

It is estimated that about 2 billion people globally have been exposed to hepatitis B viral (HBV) infection [1, 2]. Nearly 3 million people are chronically infected and are at risk for serious morbidity and death [3]. Therefore, hepatitis B poses a major public health threat to the world as well as being the deadliest liver infection [4]. Approximately two people die every minute and about three new people get infected with hepatitis B at the same time [5]. The increased infection rate as well as the threat to global public health made the World Health Organization (WHO) formulate a global viral hepatitis strategy in 2016 that is targeted at eliminating HBV infection by the year 2030 [6]. The hepatitis B virus is highly infectious since it is able to survive outside the body of its host for about seven days at room temperature [3]. This is the major reason why HBV infection is highly contagious. This makes people with certain occupations have a higher risk of contracting the virus. Health care workers (HCWs) have about a four-fold risk of contracting HBV infection compared to the general adult population. This means HCWs should be given the needed attention as far as hepatitis B is concerned. For HCWs, the risk increases with increasing employment duration [7–10]. In addition, HCWs are frequently involved in invasive procedures and as a result stand a high chance of acquiring blood-transmitted infections as they go about their duties [8].

Hepatitis B vaccination programmes organised for HCWs have resulted in the decline in the infection rate among these high-risk groups [11–13]. Such reports greatly support the assertion that hepatitis B vaccines are the safest, most available, and most effective means of preventing the infection [14, 15]. Studies have demonstrated that one needs to take all three doses of the vaccine according to the recommended schedule for the needed protection [11, 16]. This means that adherence to the primary vaccination course is essential to the success of the vaccination process.

However, other studies show that not all individuals develop the protective antibody levels (the universally recognized cut-off value of ≥10 mIU/mL) even though the usually recommended schedule may be strictly adhered to [17, 18]. Therefore, Centre for Disease Control and Prevention (CDC) recommends that, for high-risk individuals such as HCWs, a postvaccination test should be done to confirm their immunity to the infection or otherwise [19]. All such individuals with less than optimal postvaccination results should be encouraged to either take a booster dose or revaccinate for adequate protection [19].

In Ghana, there is not much data on hepatitis B postvaccination outcomes and evidence of hepatitis B vaccine efficacy among HCWs. As a result of this, vaccine nonresponders are not being identified, let alone educated, before or after exposure to the virus. This study aimed at determining the seroconversion rate and prevalence of hepatitis B viral infection among vaccinated HCWs in the Ashanti region, Ghana. Empirical findings of this study would be useful in creating awareness for especially high-risk groups to do the postvaccination testing, or otherwise it could possibly become a national policy.

## 2. Materials and Methods

### 2.1. Ethical Consideration

Ethical approval was sought from the Committee on Human Research, Publication, and Ethics (CHRPE) of the School of Medicine and Dentistry, Kwame Nkrumah University of Science and Technology (KNUST) (Reference number: CHRPE/AP 1350/21). Approval letters were obtained from all five study sites before the commencement of the study. Written informed consent was obtained from participants before their recruitment into the study. Study participants were assured of confidentiality, and the research team treated all data obtained as such. Codes were used instead of names to obscure the identity of the participants. Lastly, feedback was given to participants after the laboratory analysis on their postvaccination outcome. HBV-infected ones were helped to see clinicians. Those who had less than optimal hepatitis B surface antibody were advised to either revaccinate or take a booster dose, depending on their postvaccination results and available data.

### 2.2. Study Design and Setting

The study was cross-sectional. Both quantitative and qualitative methods were employed in gathering and analysing the data obtained from the study participants. A purposive sampling technique was used, and a total of 424 participants were recruited from the five different health facilities in the Ashanti region of Ghana. The study participants were HCWs who had taken at least two doses of the hepatitis B monovalent vaccine not less than a month prior to sampling and testing. Meanwhile, HCWs who were aware of being positive for the hepatitis B viral infection were excluded from the study.

### 2.3. Data Collection and Laboratory Analysis

After administering the questionnaire and obtaining all the necessary information including sociodemographic characteristics and participant vaccination history, about 2 ml of blood sample was collected from each study participant into a serum separator tube (Vacusera Serum Clot Activator Tube). The samples were transported in a cold box to Medilab Diagnostic Services Limited for processing, storage, and testing.

Each clotted blood sample was centrifuged, and the serum was aliquoted into cryotubes in order to avoid multiple freeze-thaw cycles and stored frozen at −20°C freezer until testing was done. During laboratory testing, aliquots of samples were completely thawed and well mixed prior to testing. The tests were done according to the manufacturer's instructions. The OneStep HBV Combo RapidCard™ Insta Test was used to determine if any of the hepatitis B viral (HBV) markers were present in the sample. The markers that were tested for were hepatitis B surface antigen (HBsAg), hepatitis B surface antibody (HBsAb), hepatitis B e antigen (HBeAg), hepatitis B e antibody (HBeAb), and hepatitis B core antibody (HBcAb). Antibody quantification (titre) of HBsAb (anti-HBs) of samples which showed positive from the qualitative test was done using Beckman Coulter Access 2 Immunoassay Analyzer. The data can be accessed online via https://figshare.com/account/articles/22270351.

### 2.4. Definition of Key Concepts

*Chronic infection* is the persistence of the hepatitis B surface antigen for at least 6 months.

*Susceptible group* involves individuals who are negative to the hepatitis B surface antigen with no surface antibodies and therefore are vulnerable to getting the infection.

*Possible recovery* includes individuals who are negative for the hepatitis B surface antigen with a positive core antibody and have not produced detectable levels of the surface antibodies.

*Recovery with immunity* refers to individuals whose surface antibodies are due to convalescence from natural infection having positive core antibodies.

*Successfully vaccinated* refers to individuals whose surface antibodies are a result of vaccination.

### 2.5. Data Analysis

The data obtained were coded and entered into Microsoft Excel 2019. All data analyses were done using GraphPad Prism 9 (GraphPad, La Jolla, CA, USA). Absolute frequency and their corresponding proportions (%) were used to summarize categorical variables. The median along with the interquartile range (IQR) was used to describe nonparametric continuous variables. Fisher's exact test and chi-square test were used to compare two and more groups, respectively. Bivariate and multivariate logistic regressions were done to assess the potential determinants of seroconversion status. Crude and adjusted odds ratios and their respective 95% confidence intervals (CI) were presented to measure the strength of association. All statistical tests in this analysis were two-tailed and *p* values <0.05 were considered statistically significant.

## 3. Results

Table 1 shows the sociodemographic and other relevant characteristics of the study population. Out of the 424 study participants, 271 (63.9%) were females and 153 (36.1%) were males. The majority (67.5%) of them were aged 20–29 years. Hospitals (50.9%, *n*/*N*  = 216/424) and schools (45.3%, *n*/*N*  = 192/424) were the commonest settings where HBV vaccinations were carried out. Medical laboratory scientists and nurses administered 40.1% and 34.4% of the HBV vaccine, respectively, to the participants of this study. The commonest vaccine administration route was intramuscular (83%, *n*/*N*  = 352/424).

**Figure 1:**
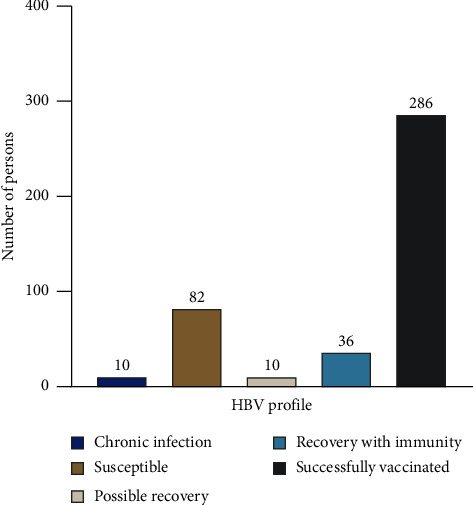
HBV status classification of vaccinated HCWs.

Figure 1 shows the HBV profile and ELISA results for the 424 study participants who had received at least two doses of the vaccine. Seroconversion (≥1 mIU/mL) and seroprotection (≥10 mIU/mL) through vaccination only among study participants were 67.5% (*n*/*N*  = 286/424) and 58.0% (*n*/*N*  = 246/424), respectively. In addition, 8.5% (*n*/*N*  = 36/424) of the study participants were seroprotected due to exposure and recovery from previous HBV infection. The overall seroconversion and seroprotection rates through vaccination together with recovery from natural infection were 76.0% and 66.5%, respectively. Interestingly, 2.4% (*n*/*N*  = 10/424) were positive to both HBsAg and HBcAb, indicative of HBV infection.

Age, the number of doses received, taking a booster dose, and keeping a vaccination record card were significant factors (*p* < 0.05) influencing seroconversion status (Table 2). The 20–29 age group recorded the highest (73.4%) seroconversion while the ≥40-year group recorded the least (21.4%). Seroconversion was observed to increase in participants as the number of doses of vaccine received increased from two (37.2%) to three (68.8%) and above three (87.8%) (*p* < 0.0001). Only 6.4% (*n*/*N*  = 27/424) had received a booster dose and 88.9% (*n*/*N*  = 24/27) of them were seroconverted. Those who had documentation on their HBV vaccination recorded significantly higher (74.5%, *n*/*N*  = 158/212) seroconversion rate than those who did not (60.4%, *n*/*N*  = 128/212) (*p* = 0.0026). However, sex, the setting HBV vaccine was administered, who administered it, the route of administration, and the year since the last dose of the vaccine was received did not have a significant effect on HBV seroconversion status (*p* > 0.05) (Table 2).

Table 3 shows the bivariate and multivariate analysis of factors associated with HBV seroconversion. The number of doses of hepatitis B vaccine received and taking a booster dose were the significant factors that were found to be associated with seroconversion. According to the adjusted odds ratio, health workers who had received three doses of the HBV vaccine were 3.86 (95% CI: 1.99–7.37) times more likely to show seroconversion as compared to those who received two doses. Likewise, those who had taken a booster dose were 4.09 (95% CI: 1.38–17.57) times more likely to develop hepatitis B antibodies than those who had not.

## 4. Discussion

This study observed seroconversion and seroprotection through vaccination (as well as recovery from natural infection) to be 76.0% and 66.5%, respectively. The prevalence of HBsAg was 2.4%, and that of HBcAb was 13.2%. Seroconversion through vaccination only was 67.5% among the study participants. The current finding is comparable to a study conducted in Cameroun (64.9%) [20]. The seroprotection rate was higher than a similar study in children done in the Savanna region of Ghana [21]. That study found seroprotection rate to be 56% while the prevalence of HBsAg and HBcAb was 1.4% and 2.0%, respectively [21]. Still, the seroconversion rate recorded in this study was lower than that of a study by Obiri-Yeboah et al. in the Central region of Ghana (91%) [22]. This seemingly lower seroprotection rate observed in the present study as compared to that of Obiri-Yeboah et al. [22] could be due to differences in the study population and the time interval between the last dose and sampling for the study [23–26]. The 2.4% of participants infected with HBV could be due to vaccination failure and subsequent exposure to the infection as a result of their high-risk working environment. It could also be as a result of undetectable levels of the HBsAg in their blood at the time of prevaccination testing [27]. The findings of this study have reaffirmed the need to perform HBV postvaccination testing for high-risk persons especially health care workers who have been vaccinated to determine their seroconversion and eligibility for a booster dose, revaccination or otherwise. Interestingly, medical laboratory scientists administered the vaccines to 40.1% of the study participants. Although not licensed to vaccinate, this cadre of HCWs are frequently involved in the administration of the vaccine to most people in Ghana. This may be because they perform the prevaccination screening testing.

The age group with the least seroconversion rate was ≥40 years. This finding is in agreement with several studies that have shown that aging correlates negatively with vaccine immune responses [28–31]. This phenomenon can be attributed to the immune system of the elderly undergoing remodification and producing increased dysfunctional memory cells and fewer naive cells [32]. Sex, setting HBV vaccine was administered, and the route of administration did not affect seroconversion status significantly, as also reported by other studies [22, 33]. However, keeping a vaccination record card was significantly associated with seroconversion. The possible explanation for this could be that these groups of people are more likely to comply with dose intervals and will be aware of the exact date to go for their next dose. Again, having authentic documentation on the vaccination may be indicative of the vaccine being received from a qualified or trusted source.

Those who received three doses of the vaccine were 3.7 times more likely to show seroconversion than those who had taken only two doses. According to Ghorbani et al. [34], taking two doses of the HBV vaccine produces immunity for only five years. Similar to this study, Van Der Meeren et al. [35] found high HBV antibody titre levels in adolescents aged 15-16 years who took all three doses of HBV vaccine in their infancy. The subject of booster doses after successful completion of the HBV primary vaccination course is a controversial one. Nonetheless, this study reported that those who had taken a booster dose were 4.1 times more likely to develop HBV antibodies than those who had not received booster doses. Although this is expected, the smaller proportion of the participants who had taken booster dose compared to the larger sample of those who had not taken it could have accounted for the four-fold difference. Meanwhile, several researchers have reported that booster doses are not required in healthy persons who have completed the full course of vaccination [4, 23, 25, 36–41]. However, other studies have suggested the need for booster doses in immunocompromised and endemic populations [42, 43].

As much as 24.1% of the hepatitis B vaccine recipients in the present study did not develop antibodies. This is higher than the estimated 5–10% who may be nonresponsive after completing two full series of the vaccination course [44]. This is probably because the majority of the study participants (about 84%) had taken their last dose for more than one year prior to sampling. As a result, this fraction of no antibody may not be a true reflection of their immune status since the waning of the antibodies with time may contribute to this [25, 26]. From this study, most of the participants had completed only one full series of vaccination. Other factors that could account for the high vaccine unresponsive rate observed in this study could be chronic illness and obesity [45]. Meanwhile, the postvaccination testing which is recommended by the CDC to be done one to two months after the vaccination course was not fully satisfied in this study [19]. This is because it has been reported that, as time elapses, the antibody levels may decline, leaving memory cells that may not be detectable by the method employed in this study [16, 24, 25, 36, 46]. Therefore, the findings from this study regarding 76.0% seroconversion and 66.5% seroprotection may not truly assess HBV vaccine efficacy. Also, this study reported the prevalence of HBsAg among vaccinated HCWs to be 2.4%, which is higher than the 1.0% reported in the Cape Coast Metropolis, Ghana [22]. A recent systematic review and meta-analysis conducted in Ghana estimated the seroprevalence of HBV as follows: 14.30% in the adolescent population and 8.36% in the adult population [47]. The lower HBV prevalence observed (2.4%) despite high-risk study participants in comparison with that of the general Ghanaian adult population (8.4%) reported could be attributed to the efficacy and the effectiveness of hepatitis B vaccines. Therefore, the present study affirms the need for HCWs to undergo a supervised, complete HBV vaccination since those vaccinated recorded fewer infections compared to the general Ghanaian population. This suggests the effectiveness of vaccination in reducing prevalence of HBV infection among people especially high-risk ones.

This study has some limitations. The HCWs self-reported their vaccination history; therefore, there could be recall bias in this study. Also, information was not obtained on the cadre of the participant HCWs, chronic illness, smoking, and obesity which are all factors that could impact the seroconversion status. Moreover, the data on the age at which the participants were vaccinated were not taken. Again, the participants had taken their last vaccine dose at varied times which could affect the seroprotection rate recorded. However, the methodology employed in this study was relevant in revealing whether a participant had immunity through vaccination or a recovery from natural infection. This study was able to identify HCWs who were infected with HBV even though they claimed they had taken the vaccine.

## 5. Conclusion

Findings of the study suggest that it is one thing to get vaccinated and another thing to get immunized. The majority of the vaccinees had protective surface antibodies to the infection in their blood. The study underscores the need for high-risk individuals to do postvaccination testing after HBV vaccination to confirm immunity or otherwise after a supervised primary hepatitis B vaccination course. Postvaccination serological testing should be done for all HWCs to confirm immunity and to reduce their chances of acquiring HBV infection. This study has given an insight into the state of hepatitis B postvaccination outcomes of the healthcare workers in the Ashanti region of Ghana.

## Figures and Tables

**Table 1:** Sociodemographic and other relevant characteristics of the study population.

Variable	Median/frequency (*n*)	Percentage (%)/interquartile range
Age, years (*N* = 424)		
Median	27	24–31
<20	8	1.9
20–29	286	67.5
30–39	116	27.4
>40	14	3.2
Sex		
Male	153	36.1
Female	271	63.9
Location HBV was administered		
School	192	45.3
Hospital	216	50.9
Other	16	3.8
Who administered HBV		
Doctor	13	3.1
Nurse	146	34.4
Pharmacist	8	1.9
Medical laboratory scientist	170	40.1
Phlebotomist	5	1.2
Do not remember	82	19.3
Route of administration		
Intramuscular	352	83.0
Subcutaneous	25	11.1
Do not know	47	5.9

**Table 2:** Association between demographics and other characteristics of HCWs and seroconversion status.

Variable	Seroconversion		*p* value
	Yes, *n* (%)	No, *n* (%)	
Age, years (*N* = 424)			**<0.001**
<20	4 (50.0)	4 (50.0)	
20–29	210 (73.4)	76 (26.6)	
30–39	69 (59.5)	47 (40.5)	
≥40	3 (21.4)	11 (78.6)	
Sex			0.914
Male	104 (68.0)	49 (32.0)	
Female	182 (67.2)	89 (32.8)	
Setting HBV was administered			0.354
School	135 (70.3)	57 (29.7)	
Hospital	141 (65.3)	75 (34.7)	
Other	9 (56.3)	7 (43.7)	
Who administered HBV			0.726
Nurse or doctor	110 (69.2)	49 (30.8)	
Medical laboratory scientist	116 (68.2)	54 (31.8)	
Other	9 (69.2)	4 (30.8)	
Do not remember	51 (62.2)	31 (37.8)	
Route of administration			0.604
Intramuscular	241 (68.5)	111 (31.5)	
Subcutaneous	16 (64.0)	9 (36.0)	
Do not know	29 (61.7)	18 (38.3)	
Number of doses received			**<0.001**
2	16 (37.2)	27 (62.8)	
3	234 (68.8)	106 (31.2)	
>3	36 (87.8)	5 (12.2)	
Years since last vaccination			0.249
<1	31 (88.6)	4 (11.4)	
1–4	103 (66.9)	51 (33.1)	
5–10	144 (65.5)	76 (34.5)	
>10	8 (53.3)	7 (46.6)	
Booster taken			**0.018**
Yes	24 (88.9)	3 (11.1)	
No	262 (66.0)	135 (34.0)	
Had vaccination record card			
Yes	158 (74.5)	54 (25.5)	**0.003**
No	128 (60.4)	84 (39.6)	

Bold values indicates they were used for the statistically significant variables.

**Table 3:** Bivariate and multivariate analyses of some factors associated with seroconversion status among HCWs.

Variable	Crude odds ratio	95% CI	Adjusted odds ratio	95% CI
Age, years (*N* = 424)				
<20	Reference			
20–29	2.76	0.64–11.95	2.86	0.66–12.43
30–39	1.47	0.33–6.49	1.49	0.34–6.58
≥40	0.27	0.04–1.75	0.28	0.04–1.78
Sex				
Female	Reference			
Male	1.04	0.68–1.59	0.90	0.58–1.40
Location HBV was administered				
Hospital	Reference			
School	1.26	0.83–1.92	0.91	0.57–1.45
Other	0.89	0.32–2.69	1.16	0.38–4.03
Who administered HBV				
Nurse or doctor	Reference			
Medical laboratory scientist	0.96	0.60–1.53	1.09	0.61–1.95
Other	1.00	0.31–3.84	1.17	0.34–4.71
Don't remember	0.73	0.42–1.29	0.73	0.42–1.29
Route of administration				
Intramuscular	Reference			
Subcutaneous	0.82	0.36–1.99	1.04	0.36–3.07
Do not know	0.74	0.40–1.41	0.64	0.34–1.31
Number of doses received				
2	Reference			
3	**3.73**	**1.95–7.34**	**3.86**	**1.99–7.37**
Years since last vaccination				
<1	Reference			
1–4	**0.26**	**0.10–0.74**	**0.24**	**0.08–0.61**
5–10	**0.24**	**0.09–0.66**	**0.24**	**0.09–0.66**
>10	**0.15**	**0.04–0.70**	**0.15**	**0.04–0.70**
Booster taken				
No	Reference			
Yes	**4.12**	**1.41–17.56**	**4.09**	**1.38–17.57**
Had vaccination record card				
Yes	Reference			
No	**0.52**	**0.34–0.79**	**0.57**	**0.38–0.81**

CI, confidence interval; crude odds ratios and adjusted odds ratios significantly higher or lower than 1 are shown in bold.

## Data Availability

The data can be accessed online via https://figshare.com/account/articles/22270351.
